# Visualizing and clustering high throughput sub-cellular localization imaging

**DOI:** 10.1186/1471-2105-9-81

**Published:** 2008-02-04

**Authors:** Nicholas A Hamilton, Rohan D Teasdale

**Affiliations:** 1 ARC Centre of Excellence in Bioinformatics, The University of Queensland, Brisbane, Queensland 4072, Australia; 2 Institute for Molecular Bioscience, The University of Queensland, Brisbane, Queensland 4072, Australia; 3 Advanced Computational Modelling Centre, The University of Queensland, Brisbane, Queensland 4072, Australia

## Abstract

**Background:**

The expansion of automatic imaging technologies has created a need to be able to efficiently compare and review large sets of image data. To enable comparisons of image data between samples we need to define the normal variation within distinct images of the same sample. Even with tightly controlled experimental conditions, protein expression can vary widely between cells, and because of the difficulty in viewing and comparing large image sets this might not be observed. Here we introduce a novel methodology, iCluster, for visualizing, clustering and comparing large sub-cellular localization image sets. For each member of an image set, iCluster generates statistics that have been found to be useful in distinguishing sub-cellular localization. The statistics are mapped into two or three dimensions such as to preserve distances between the statistics vectors. The complete image set is then visualized in two or three dimensions using the coordinates so determined. The result is images that are statistically similar are spatially close in the visualization allowing for easy comparison of images that are similar and distinguishment of dissimilar images into distinct clusters.

**Results:**

The methodology was tested on a set of 502 previously published images containing 10 known sub-cellular localizations. The clustering of images of like type was evaluated both by examining the classes of nearest neighbors to each image and by visual inspection. In three dimensions, 3-neighbor classification accuracy was 83.2%. Visually, each class clustered well with the majority of classes localizing to distinct regions of the space. In two dimensions, 3-neighbor classification accuracy was 68.9%, though visually clustering into classes could be readily discerned. Computational expense was found to be relatively low, and sets of up to 1400 images visualized and interacted with in real time.

**Conclusion:**

The feasibility of automated spatial layout to allow comparison and discrimination of high throughput sub-cellular imaging has been demonstrated. There are many potential applications such as image database curation, semi-automated interactive classification, outlier detection and reference image comparison. By allowing the observation of the full range of imaging data available using modern microscopes these methods will provide an invaluable tool for cell biologists.

## Background

The sequencing of numerous genomes and subsequent identification of the encoded proteome has created the need for large-scale systematic approaches to understand the functions of the tens of thousands of proteins at the cellular level [1,2]. High-throughput automated fluorescent microscope imaging technologies enable the experimental determination of a protein's sub-cellular localization and its dynamic trafficking within a range of cellular contexts. These approaches generate vast numbers of images including multiple fluorophores for cells under a variety of experimental conditions [3,4]. The desire and the ability to carry out high-throughput screenings of protein localization and trafficking for applications such as drug discovery [4,5] is leading to a rapid growth in cell images in need of analysis on a scale comparable to that of the genomic revolution [6]. Further, microscope technology has developed to the point that is now possible to do whole proteome imaging for sub-cellular localization [7].

To deal with the scale of the data becoming available automated annotation, analysis, comparison, classification and storage of cellular images is essential [8]. In this respect, image statistics have proved to be of great utility. Statistical measures may be used to generate a numeric vector for each cell image, and have a wide range of applications such as automated sub-cellular localization classification [9-12], image clustering [13], representative image selection [14,15] and statistical differentiation of protein localization under varying experimental conditions [16].

While high throughput imaging and automated analysis techniques are extremely useful, they suffer by removing the trained researcher from actually examining the majority of the images. Protein expression is rarely a simple process. For a given set of experimental conditions, there may be hundreds or thousands of cells on the plate, each with a distinct pattern of expression, particularly within tissue samples. Some proteins may localize to multiple locations in a single cell in varying amounts. In this way sub-cellular localization classification is distinct from many other image classification problems for which the classes have clearly defined boundaries: an image containing an everyday object might be of a car or a cow, but it can't be 20% cow and 80% car. Further, many of the structures that exist in a cell are only just beginning to be described. Hence automated classification, while very useful in a range of problems, is limited in that the classes into which to classify are predefined, the classes can "mix", and cells can do unexpected things.

One approach to avoiding the use of fixed classes and make sense of large image sets is to use image statistics to cluster the images [13]. The difficulty here is that there is a wide range of clustering methods, many of which can produce quite different clusters according to the assumptions of the algorithm. But the question that is fundamentally important to the cell biologist is: "*are the clusters biologically meaningful and what is the defining property of the cluster?"* Without a trained biologist examining each image within each cluster it is impossible to know.

In a typical sub-cellular localization experiment a single well can be imaged in perhaps two minutes and may contain over a thousand cells. However, only a few tens of cells are examined in detail, and a "typical" image published or stored in a database with a sub-cellular localization classification. To be able to fully exploit the full range of cellular imaging data that is becoming available and utilize the expert knowledge of the researcher, new techniques are required.

**Figure 1:**
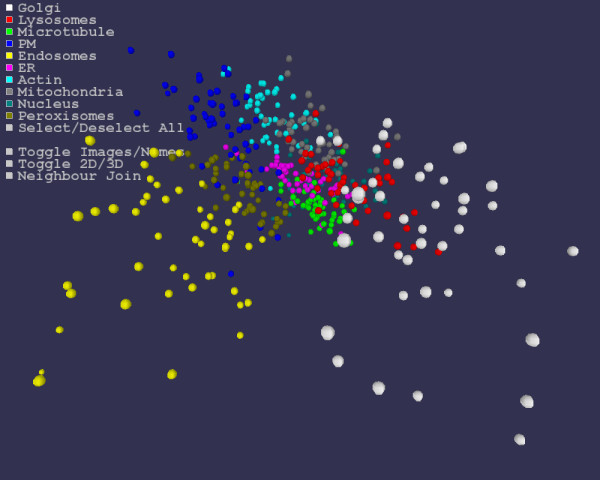
**Mapping image statistics into 3 dimensions**. For each of the 502 sub-cellular localization images, Haralick statistics and TAS measures are generated. The statistics vectors for the image set are Sammon mapped into 3 dimensions and visualized as shown with a sphere for each image. Each color represents a different sub-cellular localization. Each sub-cellular localization clusters well under the mapping, though there is some spatial overlap between classes such as mitochondria and endoplasmic reticulum. See also Additional file 1.

**Figure 2:**
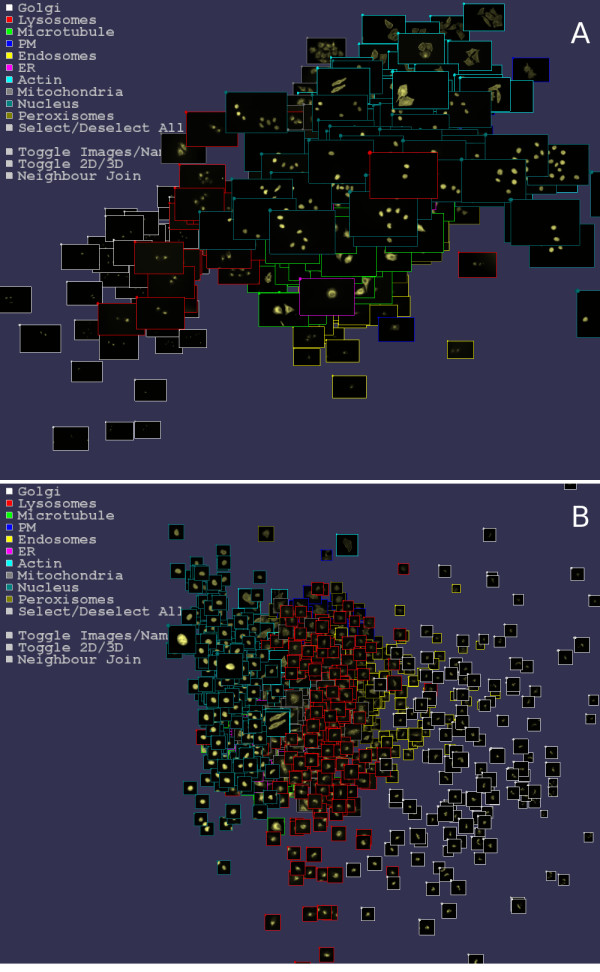
**Visualizing the image set in 3 dimensions**. (A) The 502 endogenous images visualized in 3 dimensions with coordinates determined from Sammon mapping Haralick and TAS measures. (B) 1407 cell images automatically cropped from the 502 images and visualized in 3 dimensions with coordinates determined from Sammon mapping Haralick and TAS measures. In both (A) and (B) images of each sub-cellular localization are strongly regionalized. In (A) note the clearly nuclear image center right with a lysosome (red border) classification. Possibly the DAPI nuclear image was incorrectly named when the image set was collected. Within the visualizer the user may zoom in/out, rotate, translate, click to select image classes to show/hide, show image names and visualize nearest neighbors (Figure 3). See also Additional file 2 and a simplified java script version available at [17].

Here we describe a novel methodology for visualizing large image sets with the aim of allowing easy comparison and differentiation of images of sub-cellular localization. The question we wish to answer is whether images of sub-cellular localization can be arranged in two or three dimensions in such a way that similar images are spatially close and dissimilar images are distant, thus allowing a cell biologist to view and draw more refined conclusions about the range of a protein's localization. Towards this end we have developed a prototype system, iCluster, for image statistics generation and large image set visualization. The essential idea is to use image statistics which have been shown to be useful in distinguishing sub-cellular localizations, map those statistics into 2 or 3 dimensions in such a way as to preserve the distance relationships between image statistics vectors, and visualize the images at the coordinates so determined in 2 or 3 dimensions. The result is a system within which the user can navigate, interact with and compare large biological image sets (see Figures 1 and 2, Additional files 1 and 2, web reference [17]). In the following we describe the methodology in detail, test its ability to cluster like images both visually and using quantitative nearest neighbor measures, and conclude with some potential applications in high throughput sub-cellular imaging.

## Methods

### Image data sets collection and computational resources

An image collection was previously created [10,18] for 10 sub-cellular organelles for which an endogenous protein or feature of the specific organelle was detected with a fluorescent antibody or other probe. For details of fluorescent microscopy imaging techniques see [19]. Each image was accompanied by an additional image of the cells counterstained with the DNA specific dye 4',6-diamidino-2-phenylindole (DAPI), which highlights the location of the nucleus of every cell in the image. In addition, the DAPI image was reviewed to exclude images that contained one or more cells not in interphase. Each organelle set consists of 50 localization images and 50 DAPI counterstained images. The DAPI counterstained images we not used for the purposes of this paper. All images were of fixed HeLa cells, taken at 60× magnification under oil immersion. The images are 8 bit grayscale, 768 by 512 pixels, each containing up to 13 cells. The complete image set is available for download from the LOCATE website [1,20].

All software described herein was run on an Intel Core Duo 2 T5600 notebook with nVidia GeForce Go 7900 GS graphic card under the Fedora Core 8 Linux operating system.

## Results

### Algorithm

#### Overview

For each image in a collection a vector of statistics is generated. The aim is then to map the set of statistics vectors of the images into 2 or 3 dimensions such that the distance between points of the image set are preserved as well as is possible. Once such a mapping has been found each image of the image set is then associated with a point in 2 or 3 dimensions, and the results visualized.

#### Image Statistics

A wide variety of classes of image statistics have been tested for their ability to distinguish images of sub-cellular localization. Conrad et al. (2004) tested four hundred and forty-eight different image features and applied a variety of feature reduction and machine learning methods [9]. Of those tested, Haralick texture measures [21] were found to give the best performance. Our own work [10,18] and the work of Murphy lab [15] have also shown the utility of Haralick texture measures in analysis of sub-cellular imaging. Subsequently, our group introduced threshold adjacency statistics (TAS), and found that these statistics in combination with machine learning methods could provide comparable classification accuracy (up to 95%) to the Haralick measures while being at least an order of magnitude faster to calculate [10]. Hence in the iCluster system, for each image in a set, two classes of statistics are generated: 27 threshold adjacency statistics (TAS) calculated as described in [10] and 23 Haralick texture measures as described in [10,18].

Note that each image may contain multiple cells and that statistics are calculated on the complete image without selecting and cropping individual cells from the image. Experiments in which a simple automated system was used to cropping individual cells [18] produced similar results to those described in herein (data not shown). Each image statistic is normalized by subtracting the mean for that statistic across the image set and dividing by the standard deviation.

#### Sammon Mapping

Once image statistics have been calculated for a complete image set, the vector of statistics for a given image then corresponds to a point in a high dimensional space. In the case of the TAS the space has dimension 27. The aim is then to find a map from 27-dimensional space to 2 or 3-dimensional space in such a way that the Euclidean distance between the points corresponding to the images are preserved as closely as possible under the mapping. That is, if the distance between a pair of image points in 27 dimensional space is *d*, then the distance between the corresponding points in 2 or 3-dimensions under the mapping should be approximately *d*. To do this Sammon mapping is employed [22]. An initial random mapping from the high to low dimensions is chosen and is modified using a steepest descent algorithm to minimize the distance error

$$E=\frac{1}{\sum_{i<j}d_{ij}^{*}}\sum_{i<j}^{N}\frac{{\left(d_{ij}^{*}-d_{ij}\right)}^{2}}{d_{ij}^{*}}\;$$

where *d*_ij_* is the distance between vectors *i *and *j *in the high dimensional space, and *d*_*ij *_the distance in the low dimensional space. The *stress *of the mapping is then the distance error for the final mapping. Low stress values correspond to a good mapping. Note that the choice of error function means that Sammon mapping emphasizes preservation of local distances over the long range ones. For the current application local distances are the most important for comparison of similar images, while for dissimilar images all that we require is that they be spatially distance from one another.

Self organizing maps were also briefly considered as a mapping method, but these can be computationally expensive. Principle component analysis (PCA) was also investigated, but found to give poor spatial clustering of classes (data not shown). However, principle components may be used to initialize the low dimensional coordinates for the Sammon mapping [23,24] and was found to decrease convergence time by a factor of 2.

Sammon mapping may use a distance measure other than the Euclidean distance. A commonly used measure is the Mahalanobis distance which takes into account correlations between features. However, Chen and Murphy [13] when comparing Euclidean and Mahalanobis distance for clustering sub-cellular localisation image statistics found the Euclidean distance to be a more stable measure. For this reason, as well as computational expense, Euclidean distance is employed in the iCluster pipeline.

#### Visualization and Interaction

We describe here the visualizer in 3 dimensions. Two dimensions is essentially the same and the user may easily switch between the two via mouse or keyboard interaction. Initially the user is presented with a 3 dimensional environment in which each image is represented by a sphere, the coordinates of the sphere being given by the mapping of that image's statistics under the Sammon map (Figure 1 and Additional file 1). If images are of known classes, such as mitochondria or nucleus, the user may supply a text file where each line gives an image name and a class. This is used in the visualization to distinguish images classes by color and to generate a figure legend. If no image classes have been given, the spheres are of uniform color. It should be noted that the known classes of the images are used only for the visualization and neighbor testing and are not utilized by the Sammon mapping.

Navigation is controlled via either keyboard or mouse. The user may rotate, zoom in or out, or translate vertically. The radius of the spheres is also readily changed to prevent spheres from obscuring others. Either the images themselves or the image names may be selected to be drawn at the 3 dimensional locations associated with each image (Figure 2 and Additional file 2). The apparent size of the images displayed is easily changed through mouse or keyboard. If image classes have been supplied, the images or names of a given class or classes may be selected to be visualized by clicking on the corresponding legend boxes. This enables easy visual comparison of particular classes of interest. Snap shots images of the current view may also be saved to disk.

**Figure 3:**
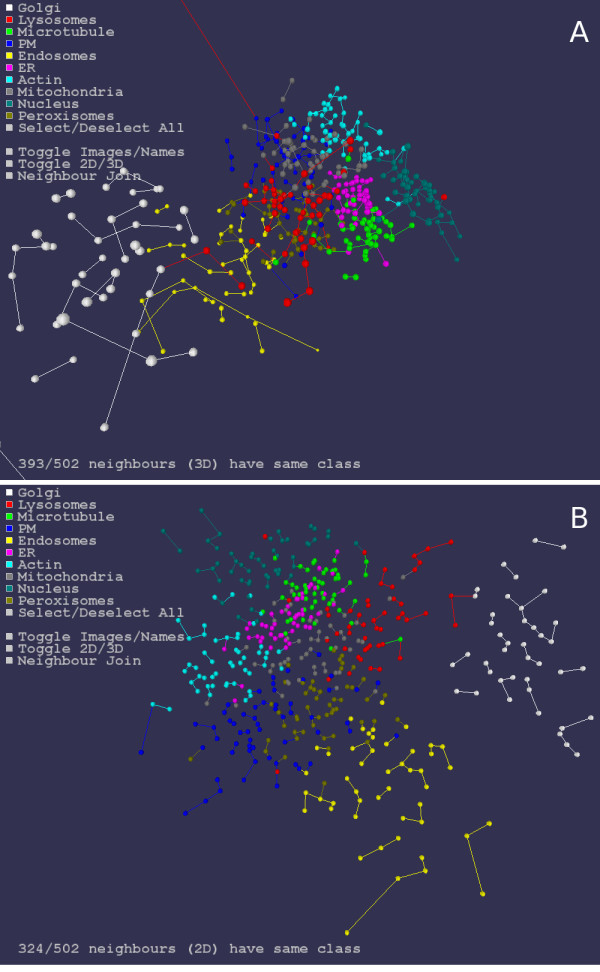
**Nearest neighbors in 2 and 3 dimensions**. Nearest neighbors for the Sammon mapped Haralick and TAS statistics of the 502 images are shown in 2 (B) and 3 (A) dimensions. Nearest neighbors using the Euclidean distance measure between the images statistics vectors, rather than the Euclidean distance in 2 or 3 dimensions, may also be shown in the visualization. See also Supplementary Movie 3.

The nearest neighbors of the images may also be visualized (Figure 3 and Additional file 3). For each sphere in 3-space corresponding to an image, a line is drawn to the closest sphere (image) in the 3-space. The nearest neighbors using the Euclidean distance between the original image statistics vectors, rather then the 3-space distance may also be shown. If image classes have been supplied, the number of images whose class is the same as that of their nearest neighbor is shown. Images may also be viewed while nearest neighbors are visualized.

### Testing

#### Image Set

The utility of the application of Sammon mapping sub-cellular localization image statistics was tested on a set of 502 images for which an endogenous protein or feature of a specific organelle was detected with a fluorescent antibody or other probe (see Methods above). The localizations fall into 10 classes: nucleus, cytoplasm, endoplasmic reticulum, Golgi, plasma membrane, endosome, lysosome, peroxisome, mitochondria, actin cytoskeleton and microtubule, with approximately 50 images per class. Each image contains up to 13 cells. According to user preference, the iCluster prototype can either use each image as is, or may apply a simple cell selection and cropping scheme as used in [10]. The scheme involves calculating a threshold, *t *= *μ-0.9σ*, where *μ *and *σ *are the mean and standard deviation of the intensities of the pixels in the image whose intensity are in the range 30 to 250 (255 being the maximum intensity). Cells then correspond to the large above threshold regions, and are selected and cropped. For the majority of the current paper the methods are applied to un-cropped images. The image set is publicly available and further information may be found in [10,18].

#### Statistics and Sammon Maps

Three sets of statistics were tested: TAS alone; Haralick texture measures alone; and TAS combined with Haralick texture measures. For each of the three sets of statistics, Sammon maps were constructed into 2 and 3 dimensions.

#### Nearest Neighbors

A common technique for testing how well a mapping between dimensions preserves the spatial distance relations between the objects of interest is to consider nearest neighbor classification [25]. Each object has a known classification, in this case the sub-cellular localization. In a leave one out nearest neighbor classifier, each object is assigned a computed classification given as the true classification of its nearest neighbor, nearest being defined by a measure such as Euclidean distance. The *classification accuracy *of the nearest neighbor classifier is then the percentage of objects for which the true and computed classifications are identical. A minor variation is to use a voting system for the *k nearest neighbors*, for some integer k, to produce a computed classification for each object.

Nearest neighbor classification is useful in that it gives a measure of how well classes separate and locally cluster. If a class forms a cluster disjoint from others, the nearest neighbor classification accuracy will be high on that class. Conversely, if the classes are spatially mixed, the classification accuracy will be low. It may be that a given class is comprised of two or more disjoint clusters. However, if there is little spatial overlap between distinct classes, the neighbor classification score will remain high for such cases.

Results for the 502 sub-cellular localization images of nearest neighbor classification for the image statistics and the Sammon mappings of those statistics into 2 or 3 dimensions are given in Table 1. One neighbor and three neighbor classification accuracies are then given for: unmapped (nearest neighbors using Euclidean distance on the image statistics); 3D (nearest neighbors using Euclidean distance on the image statistics Sammon mapped in 3 dimensions); and 2D (similarly in 2 dimensions). See Figure 3 and Additional file 3 for visualization of nearest neighbors.

**Table 1:** Nearest neighbor classification accuracies for the 502 images of 10 sub-cellular localizations.

	TAS+Haralick			TAS			Haralick		
Statistics Dimension	Unmapped	3D	2D	Unmapped	3D	2D	Unmapped	3D	2D
1-neighbor classify	93.2%	79.1%	64.5%	91.8%	77.5%	59.6%	87.1%	68.3%	54.0%
3-neighbor classify	94.0%	83.2%	68.9%	90.6%	80.7%	64.3%	85.5%	70.9%	60.6%

Three types of image statistics vectors are tested: TAS with Haralick; TAS alone; Haralick alone. For a given image, the class of the image is compared to that of its nearest neighbors, nearest being defined using the Euclidean distance between the statistics vectors (unmapped), the statistics Sammon mapped into 3 dimensions (3D) and 2 dimensions (2D). Tests in which 1407 individual cells were automatically selected and cropped from the 502 images gave similar (in fact slightly higher) classification accuracies as those in the table (data not shown).

It can be seen that neighbor and 3-neighbor classification accuracies using the unmapped statistics are remarkably high, with an accuracy of up to 94% (TAS+Haralick, 3-neighbor). Previously, a 5-fold cross validation classification accuracy of 98.2% was obtained in [10] using a support vector machine trained on TAS and Haralick statistics. Even the lowest unmapped statistics accuracy of 85.5% (Haralick, 3-neighbor) shows that the image classes are grouping well. This is in accordance with the results of Murphy lab showing the utility of high dimensional images statistics in the clustering of sub-cellular localization images [11,13].

Nearest neighbor classification accuracies are lower in two and three dimensions for all classes of statistics. The combination of TAS with Haralick statistics gives the best results with 3-neighbor accuracies of 83.2% (3D) and 68.9% (2D). The results of TAS statistics alone are similar, though lower, with 3-neighbor accuracies of 80.7% (3D) and 64.3% (2D).

Table 2 lists nearest neighbor classification accuracies on each sub-cellular localization class using TAS together with Haralick statistics and their mappings into two and three dimensions. It can be seen that the nearest neighbor classification using the unmapped statistics vectors are overall very high, with only the peroxisome and endoplasmic reticulum classes having accuracies below 90%. In three dimensions the results are more variable, ranging from 97.9% (Golgi) to 64.8% (peroxisome). In two dimensions, mitochondria are poorly classified (38.5%), though Golgi retains a high accuracy of 97.9%. Broadly, higher accuracy in the unmapped statistics corresponds to higher accuracy in 2D and 3D neighbor classification, though the plasma membrane is an exception.

**Table 2:** Nearest neighbor classification accuracies for the 10 sub-cellular localizations of the 502 images.

Organelle	TAS+Haralick (unmapped)	3D	2D
Nucleus	94.1%	92.2%	85.1%
Endoplasmic Reticulum	86.8%	72.2%	48.1%
Golgi	100%	97.9%	97.9%
Plasma Membrane	97.9%	77.4%	61.8%
Endosome	94.0%	81.1%	77.6%
Lysosome	92.3%	76.1%	63.3%
Peroxisome	86.2%	64.8%	53.2%
Mitochondria	93.8%	73.5%	38.5%
Actin Cytoskeleton	97.9%	82.4%	73.7%
Microtubules	91.8%	75.0%	48.9%

The nearest 1-neighbour classification accuracies for each sub-cellular localization are shown for the TAS combined with Haralick measures and their Sammon mappings in 3 and 2 dimensions.

The reduction in classification accuracy in two and three dimensions is reflected in the stress of the Sammon mappings. Mapping the combined TAS and Haralick statistics into three dimensions had a stress value E = 0.00569, while mapping into two dimensions gave E = 0.0148. Hence more distortion of the pair wise distances was required in the mapping to two dimensions. Mappings of TAS only and Haralick only gave similar 3D and 2D stress values to the combined statistics (data not shown).

It is worth noting that image classification is a well studied area with several standard measures developed for the evaluation of automated classification [26-28]. However, the problem we aim to solve is not classification, but to place images in such a way that similar images are spatially close. Hence the measure of whether nearest neighbors are of the same class is an appropriate choice for our study.

#### Visualization

While neighbor classification gives one measure of how well images of a given class are clustering, it is not the whole story. When viewed in three dimensions, the spatial clustering into classes is apparently very strong (Figures 1 and 2, Additional files 1 and 2). Examined individually, each image class appears to locate to a particular region of the space. Classes such a microtubule and endoplasmic reticulum appear tightly localized. Other classes such as Golgi and endosome are more widely dispersed, though still have their own region largely separate from the other classes. Of the sub-cellular localizations, the peroxisome images are the ones that appear to be most spatially mixed with other classes. This is reflected in the peroxisome having the lowest nearest 3D neighbor classification rate (64.8%). There is also some spatial mixing between the endoplasmic reticulum and microtubule classes. The results on these classes are as might be expected since they can be visually quite similar and have previously been observed to be difficult to distinguish using image statistics [10].

While there is some spatial overlap of classes, it is relatively easy to identify by eye a region as belonging to a given class. It should be emphasized that the aim here is not to construct a near perfect nearest neighbor classifier in three dimensions, but rather to cluster visually similar images in such a way as to allow easy comparison and distinguishment across large image sets. As long as an image is relatively close to where it "should" be, the human eye is more forgiving of slight misplacements than a neighbor classifier.

#### Computational Expense

For the test set of 502 images, Haralick statistics took approximately 1600 seconds to calculate, while TAS took 78 seconds. TAS have previously been shown to be fast to calculate while providing high classification accuracy using machine learning techniques [10]. The calculation of the Sammon maps of the combined TAS and Haralick measures into two and three dimensions took approximately 15 and 70 seconds, respectively. Calculation of Sammon maps of TAS statistics alone into two and three dimensions took approximately 35 seconds each. Hence the use of Haralick measures significantly increases the computational expense while only improving the nearest neighbor classification accuracy by a few percent (in combination with TAS). Visually, in three dimensions, whether TAS or TAS combined with Haralick statistics are used in the Sammon mapping appears to make little difference (data not shown).

Applying a simple cell selection scheme to select 1407 cells from the 502 images (see Figure 2(B)), the time to find and crop cells was 44 seconds, calculation of TAS took 61 seconds, and the Sammon maps approximately 450 seconds each. Hence the computational expense of Sammon mapping increases significantly with the number of images, while smaller image sets can be processed much faster. Tests on a set of 100 images (uncropped) using TAS measures took in total 15 seconds to calculate the statistics, both 2D and 3D Sammon mappings, and start the visualizer (data not shown). Currently, we are re-implementing the Sammon mapping in Java, and preliminary tests show that a Sammon map may be generated for 1407 images in around 100 seconds. Hence from image set to visualisation takes around 2 minutes for such a set.

Visualization was performed on a notebook computer, as were the statistics generation and Sammon map calculations. Visualizations of up to 1400 images at a time have been tested (Figure 2B). At a viewer resolution of 1430 × 830, real time interaction and navigation was fluid and responsive when viewing 1400 images. To retain fluidity of interaction, the important hardware component is a dedicated graphic card with OpenGL support. However, for smaller images sets (less than 100 images) and smaller views, the visualizer will run well on most hardware. A java applet demonstration of the software available at [17] shows 100 images and does not use OpenGL support. Hence the method of visualization and interaction with image sets could be run on most commonly available systems with the addition of a recent graphic card for larger sets.

## Implementation

Image statistics were generated using the ASPiC system as described in [10,18].

To create Sammon maps, the pipeline employed MATLAB^® ^scripts written by Gavin Cawley and Nicola Talbot. The original scripts are freely available from [29]. However, our work flow used Octave [30] rather than MATLAB^®^, though the MATLAB^® ^script will run in Octave with trivial modifications.

Image visualization in two and three dimensions was programmed in processing [31]. Processing is an open source high level programming language and environment designed to enable programming and display of images, animation, and interactions.

The components of the above work flow are linked together using the Perl scripting language. Command line parameters determine which statistics are used, whether to crop individual cells from each image, whether to scale the images for visualization, and give the option of supplying a text file with classifications for each image. When the script is run in a given directory, it finds the tif format images contained within that directory and applies the complete work flow and starts the visualizer.

## Discussion

This work is placed within the context of the large literatures on automated sub-cellular image analysis, content based image retrieval (CBIR) [32-34] and biomedical image analysis and classification generally [35]. CBIR aims to index, search and match images by their content. By avoiding the use of incomplete or non-existent annotation the goal is to improve image retrieval accuracy.

Our aim, while drawing on many of the techniques of image classification and CBIR, is slightly different. Because of the complexity of sub-cellular protein expression, we do not wish to place images into pre-defined classes or match a single image against others. Rather we want to be able to visually show the images of large sub-cellular image sets in order to reconnect the cell biologist with their data. The trained human eye is still the best pattern recognition system available, and the nature of the sub-cellular image sets requires that they be examined in as much detail as possible. Further, we aim to enable the cell biologist to interact and manipulate their image sets in real time as they are obtained from the microscope or selected from a database.

We believe the approach we have outlined is unique in a number of respects. While the use of image statistics for clustering of sub-cellular imaging has previously been studied [13,34], there has not been a visualisation component to enable visual examination of the relationships between sub-cellular images. Similarly, automated representative image selection [14,15], in which an image is selected from a set of images as the one that best represents that set is an area of considerable interest. Though not shown in the figures here, iCluster will calculate a representative image for an image class as the closest image to the centroid of the image statistics, and hence display a representative image for each image class, either embedded within the complete image set or with just the representative images shown side by side on the screen. By adding a visualisation component the aim is to allow the biologist to decide whether the image so selected is truly representative in a biological sense in the context of the surrounding images.

Another feature of iCluster is its speed. Typically, image clustering and visualisation using methods such as self organising maps have been designed to be trained offline and then visualized [23]. In a typical sub-cellular localisation experiment a single microscope well may contain 1000 to 2000 cells. The speed of iCluster opens the possibility of examining image sets as they come off the microscope and getting fast feedback on the range of cells in the well.

A variety of approaches might have been taken to the visualisation of the sub-cellular image sets. One would be a hierarchical one. Much as measures of similarity between protein sequences may be used to construct a putative evolutionary tree, measures of image similarity may be used to construct a tree [13] that might then be visualised with the images as the leafs in two or three dimensions. While this may be useful for clustering, with the spatial relationship approach taken via Sammon mapping the advantage is that the distances between pairs of images represent their similarity and hence allow greater comparison. In general, spatial organization of image sets by similarity has been found useful in finding images with particular properties [36]. References [37-39] give examples of a range of image set and visualisation techniques.

## Conclusion

We have demonstrated the feasibility of visualizing large collections of microscopy images of sub-cellular localization in such a way that similar images are clustered together spatially. In testing clustering of images of known localization, it was found that while nearest neighbor classification accuracy was reduced by the Sammon mapping of image statistics into lower dimensions, the accuracy remained high enough to group and distinguish sub-cellular localizations to a high degree. It is worth reiterating that the aim was not to construct a highly accurate neighbor classifier, but to spatially group similar images to allow for easy comparison and differentiation across large image sets. Further, it has been shown that the required computations can be performed on commonly available hardware in a matter of seconds to minutes.

There are a number of potential applications and extensions of the methodology. While there would clearly be utility in being able to view and cluster a multitude of cell images from a given experiment, a perhaps more interesting application would be to distinguishing imaging under varying experimental conditions. High dimensional image statistics have previously been shown to be able to statistically differentiate subtle changes under varying experimental conditions [16]. However, while such tests can detect statistically significant difference, they give no clue as to what that difference is. With like images grouped and visualized in 2 or 3 dimensions it would be possible to observe the difference and the degree to which the images for each condition were visually distinct.

The methodology might also be used to curate large image sets for which classifications are already known. As image databases become increasingly large it becomes difficult to ascertain the reliability of the information contained within them. Errors can occur when images are initially saved to disk from the microscope, or when images and information are imported into a database. More generally human classification can be of variable accuracy and may exhibit biases because of the visual similarity of some localization classes and variability in the degree of protein localization to various locations. Large sub-cellular image databases, such as the LOCATE mammalian sub-cellular localization database [20], regularly have their image classifications reviewed for consistency and correctness. Such image sets might be visualized in the manner of Figure 2 and quickly examined for discrepancies. For classes that are visually similar, such as endoplasmic reticulum and microtubule cytoskeleton in our image set, it should be possible to see if the initial classifier had a bias towards one classification that the reviewer could then rectify.

Another use would be in curation of automated image classifications. Depending on the classifier and the image set, automated classifiers can range in accuracy anywhere from 82% to 98% with wide variations in accuracy between sub-cellular localizations. Visualizing the images and the results of machine classification in the manner of Figure 2 would allow easy curation of the results. Further, as was noted in the introduction, protein expression can be a very variable process with some proteins being localized to multiple locations in a single cell. Machine classification will usually force a single classification, and hence miss such effects. While some machine classification methods will supply confidence scores for each class, if the localization is novel or unknown to the classifier, the confidence scores may be meaningless. By giving an initial classification which can then be reviewed by an expert cell biologist to reclassify or create new classes, the rate and quality of classification could be significantly increased.

Along similar lines, another extension would be to fast user classification of image sets of unknown localization. Images could be visualized and clustered via nearest neighbors (see Figure 3) or other methods. A cluster could then be selected by the user and have classification applied to the images within it. Images that the researcher considered to be outliers of the cluster could then be selected and reclassified. As well as speed of classification, this approach would provide a significant aid to improving classification accuracy by allowing the researcher to examine similar images while considering the classification of a given image. Potentially, "gold standard" images of known localization could be included with images of unknown localization as a reference for the researcher.

Finally, the methodology described has been applied to distinguishing and clustering images of sub-cellular localization. Other image statistics, such as measures of morphology, might also be applied to cluster and visualize cellular images to distinguish other cellular properties.

In conclusion, it has been shown that image statistics and Sammon mapping are well suited to the problem of relational spatial layout of sub-cellular localization images. We would argue that the major point of automated methods for analysing sub-cellular patterns is not to avoid looking at all of the images, but rather to enable high throughput. With the vast image sets that are becoming available, visualisation techniques will be essential in ensuring that researchers can view, comprehend and draw more refined conclusions from the wealth of data becoming available.

## Abbreviations

ASPiC, Automated Subcellular Phenotype Classification; CBIR, Content based image retrieval; DAPI, 4',6-diamidino-2-phenylindole; PCA, Principle component analysis; TAS, Threshold adjacency statistics.

## Authors' contributions

NAH designed and tested the iCluster work flow and drafted the manuscript. RDT participated in the design of the study and coordination and helped draft the manuscript. Both authors read and approved the final manuscript.

## Supplementary Material

Additional file 1 Mapping image statistics into 3 dimensions. A movie showing a rotation around the data shown in Figure 1. Note that frame size and frame rates were reduced to produce acceptable movie file sizes. Click here for file

Additional file 2 User interaction with the visualization. Initially a sphere in 3 dimensions is shown for each of the 502 sub-cellular localization images (coordinates using Sammon mapped Haralick and TAS measure). Images from the mitochondria and actin cytoskeleton classes are then selected to be viewed by clicking on their legend boxes. The view is then zoomed in and rotated to view the images in detail. Images of all classes are then selected to be viewed, and a rotation around the complete image set shown. The movie finishes showing a detailed view of a peroxisome image. All interaction shown is through a standard wheel mouse. Click here for file

Additional file 3 Nearest neighbors shown 3 dimensions. A movie showing a rotation around the data shown in Figure 3(A). Initially the movie shows and rotates around the data, with the nearest neighbors using the 3D Euclidean distance then being joined. The view is then switched to showing nearest neighbors using the (high dimensional) Euclidean distance between the unmapped statistics vectors for each image. The number of images that have the same class as their nearest neighbors is shown in each case. Click here for file
